# The differential diagnosis of adrenocortical tumors: systematic review of Ki-67 and IGF2 and meta-analysis of Ki-67

**DOI:** 10.1007/s11154-025-09945-w

**Published:** 2025-01-31

**Authors:** Sofia B. Oliveira, Mariana Q. Machado, Diana Sousa, Sofia S. Pereira, Duarte Pignatelli

**Affiliations:** 1 UMIB – Unit for Multidisciplinary Research in Biomedicine; ICBAS – School of Medicine and Biomedical Sciences, University of Porto, Porto, Portugal; 2 ITR – Laboratory for Integrative and Translational Research in Population Health, Porto, Portugal; 3 i3S – Institute for Research and Innovation in Health, University of Porto, Porto, Portugal; 4 IPATIMUP – Institute of Molecular Pathology and Immunology of the University of Porto, Porto, Portugal; 5 Department of Endocrinology, Unidade Local de Saúde de São João, Porto, Portugal; 6 Faculdade de Medicina Dentária, UCP – Universidade Católica Portuguesa, Viseu, Portugal; 7 Department of Biomedicine, Faculty of Medicine, University of Porto, Porto, Portugal

**Keywords:** Adrenocortical tumors, Diagnosis, Immunohistochemistry, IGF2, Ki-67, Meta-analysis

## Abstract

Distinguishing benign from malignant adrenocortical tumors (ACT) is not always easy, particularly for tumors with unclear malignant potential based on the histopathological features comprised of the Weiss score. Previous studies reported the potential utility of immunohistochemistry (IHC) markers to recognize malignancy, in particular the Insulin-like growth factor 2 (IGF2) and the proliferation marker, Ki-67. However, this information was not compiled before. Therefore, this review aimed to collect the evidence on the potential diagnosis utility of IGF2 and Ki-67 IHC staining. Additionally, a meta-analysis was performed to assess the Ki-67 accuracy to identify adrenocortical carcinoma. The systematic review and meta-analysis were conducted according to the PRISMA guidelines. From the 26 articles included in the systematic review, 21 articles provided individual data for IGF2 (*n* = 2) or for Ki-67 (*n* = 19), while 5 studies assessed both markers. IGF2 staining was positive in most carcinomas, in contrast to adenomas. However, the different immunostaining evaluation methods adopted among the studies impeded to perform a meta-analysis to assess IGF2 diagnostic accuracy. In contrast, for the most commonly used cut-off value of 5% stained cells, Ki-67 showed pooled specificity, sensitivity and log diagnostic odds ratio of 0.98 (95% CI 0.95 to 0.99), 0.82 (95% CI 0.65 to 0.92) and 4.26 (95% CI 3.40 to 5.12), respectively. At the 5% cut-off, Ki-67 demonstrated an excellent specificity to recognize malignant ACT. However. the moderate sensitivity observed indicates the need for further studies exploring alternative threshold values. Additionally, more studies using similar approaches are needed to assess the diagnostic accuracy of IGF2.

**Registration code in PROSPERO:** CRD42022370389.

**Supplementary Information:**

The online version contains supplementary material available at 10.1007/s11154-025-09945-w.

## Introduction

Adrenocortical tumors (ACT) can be categorized as adrenocortical adenomas (ACA) and carcinomas (ACC) depending on the tumor’s biology [1]. In contrast to ACA, ACC are rare tumors, with an estimated incidence of approximately 0.5–1 cases per million people per year [2, 3]. Most of these tumors are usually very aggressive with a 5-year overall survival less than 15%, in advanced ACC [4, 5]. An accurate diagnosis is crucial for the most appropriate clinical strategy, namely adjuvant therapy and follow-up time, as well as for predicting outcomes. Currently, the determination of ACT malignancy is based on unspecific imaging characteristics and histopathological features. Preoperatively, the malignant potential of ACT is predicted by tumor size and radiological density measured in Hounsfield units (HU) on computed tomography. After tumor removal, the differential diagnosis between benign and malignant ACT is mainly based on a multiparametric system, the Weiss score, that combines nine histopathological criteria related with tumor structure, cell characteristics and tumor invasion [6–8]. A Weiss score of ≥ 3 suggests malignancy, whereas an ACT with a Weiss score of 0–2 is classified as benign [1, 6, 9] Nevertheless, a Weiss score of 2–3 presents challenges in accurately predicting the biological behavior of ACT, as tumors with this score often fall into a 'gray zone' between benign and malignant [6, 10–13]. Although this misclassification appears to be rare [10], it can result in both over- and under-diagnosis. Over-diagnosis can lead to extensive monitoring and increased costs, whereas under-diagnosis delay the timely determination of the most appropriate treatment strategy, potentially resulting in a fatal outcome due to the aggressive behavior of ACC [14].

There is an unmet need for biomarkers to accurately identify malignancy in ACT, particularly for tumors with unclear malignant potential based on the Weiss scoring. On this context, previous studies reported the potential utility of several immunohistochemistry (IHC) markers to recognize malignancy, notably the Insulin-like growth factor 2 (IGF2) and the proliferation marker, Ki-67, which have been extensively investigated in ACT [11]. Indeed, IGF2 is one of the main oncogenes implicated in ACC tumorigenesis, while Ki-67 marker has a prognostic role and is routinely assessed in clinical practice. To our knowledge, the diagnostic accuracy of IGF2 and Ki-67 has never been systematically assessed for validation. Therefore, the aim of this review was to collect the evidence on the potential diagnostic value of IGF2 and Ki-67 IHC staining to discriminate ACA from ACC. Additionally, a meta-analysis was performed to assess the accuracy of Ki-67 as diagnostic marker for ACC.

## Methods

### Protocol and registration

The present systematic review and meta-analysis were conducted according to the Preferred Reporting Items for Systematic Reviews and Meta-Analysis (PRISMA) 2020 statement and PRISMA of Diagnostic Test Accuracy (PRISMA-DTA) [15–17]. This study was submitted to the international database of prospectively registered systematic reviews (PROSPERO) and can be accessed with the registration number CRD42022370389.

### Data sources and search strategy

A systematic search was performed in three electronic databases, including PubMed, Scopus, and Web of Science, using key words and word variants for ACT, IGF2, Ki-67, IHC expression, and diagnosis. No limitations regarding publication date were applied. The last search was conducted in March 2024. The full search string used for each database is presented in Supplementary File 1. Additionally, the reference lists of eligible articles were manually searched to identify relevant studies that had not been previously retrieved.

### Study selection and criteria

After removing duplicates, two authors (SBO and MQM) screened titles and abstracts independently for eligibility, followed by full-text reading of potentially relevant studies. When necessary, a third author (SSP) was consulted in the case of disagreement.

Eligible articles included observational studies (cohort, prospective, and retrospective studies) assessing IGF2 and/or Ki-67 expression using IHC in human ACC and ACA tissues. Only studies that reported or provided sufficient data to predict the diagnostic utility of IHC markers were included.

The exclusion criteria included articles published in languages other than English, reviews, abstracts, and conference proceedings. The authors of studies whose full text could not be accessed were contacted. In case of no response in 90 days, the manuscripts were excluded. Studies reporting data regarding ACC variants (oncocytic, myxoid, and sarcomatoid variants), tumors metastasis, and tumors from pediatric patients were excluded unless the data regarding adult conventional ACC and ACA were able to be retrieved. Articles not describing the IHC technique for Ki-67 and/or IGF2 were also excluded. Additionally, studies not presenting IHC results or not allowing comparisons between ACA and ACC IHC expression were considered ineligible.

### Data extraction and synthesis

The eligible studies were divided among two authors (SBO and MQM) for independent data extraction in a cross-over manner and later reviewed by a third one (SSP). Data included study details (name of the first author, publication year, country, and study design), clinical characteristics of tumors (biological behavior, functionality, and sample size), demographic characteristics of study patients (age and sex), diagnostic criteria (clinical, imaging and pathological diagnosis), follow-up time, IHC quantification method, IHC results (IGF2 and/or Ki-67 expression and group comparisons) and diagnosis performance measures [as sensibility, sensitivity, positive and negative predictive values, positive and negative likelihood ratios, and area under the receiving operating characteristic (ROC) curve (AUC)] when reported. If two or more articles reported the same data, only the data which included a higher number of ACT was considered.

Data extracted was summarized and presented separately for each marker in Tables 1 and 2.

**Table 1:** Characteristics of the included studies regarding Insuline-like growth factor 2 (IGF2) expression in adrenocortical tumors

First author and year	Experimental design	Sub-Groups (n)	Age in years	Sex F:M	Diagnosis tool	Follow-up time	IHC analysis method	Results		
								Group Comparisons		Diagnostic accuracy
Schmitt 2006 [18]	Retrospective	ACC (17)	Mean:52.50 Range:27–75	11:5	No uniform diagnosis criteria were used	NA	Qualitative analysis	ACC	Positive ACC: 76% (13/17)	Sensitivity of 76.5% Specificity of 95.5%
		ACA (22)	Mean:45.59 Range:20–65	16:6				ACA	Negative ACC: 95% (21/22)	
Soon 2009 [19]	Prospective	ACC (23)	NA	NA	NA	NA	Semi-quantitative analysis	ACC	ACC with positive score 2–4: 78% (18/23)	AUC = 0.863
		ACA (41)	NA	NA				ACA	ACA with negative score 0–1: 100% (41/41)	
Pereira 2013 [20]	Retrospective	ACC (11)	Median:46 Range:27–59	6:5	Weiss score	NA	Morphometric computerized analysis (SA)	ACC	Mean SA ± s.e.m: 35.31 ± 1.33%	ACC vs ACAt AUC = 0.81 ACC vs ACAn AUC = 1.00 for a cut-off value of 27.11% stained area
		ACAt (20)	Median:49 Range:23–76	14:6				ACAt	Mean SA ± s.e.m: 23.90 ± 2.44%	
		ACAc (7)	NA	NA				ACAc	Mean SA ± s.e.m: 35.73 ± 1.75%	
		ACAn (13)	NA	NA				ACAn	Mean SA ± s.e.m: 17.67 ± 2.17%	
Wang 2014 [12]	Retrospective	ACC (25)	Mean:44.4 Range:18–75	14:11	Weiss score	Range:6–123 months	Semi-quantitative analysis	ACC	Elevated expression: 64% of ACC (16/25)	NA
		ACA (25)	Mean:48.6 Range:34–69	14:11		Mean: 57 months Range: 6–123 months		ACA	Negative/low expression: 72% of ACA (18/25)	
Zhu 2014 [21]	Retrospective	ACC (24)	Mean:58.21 Range:38–74	13:11	Endocrine evaluation, image examination and Weiss score	Mean: 151.5 months Range: 102–264 months	Semi-quantitative analysis	ACC	Positive ACC: 70.83% (17/24)	NA
		ACA (20)	Mean:44.95 Range:28–59	12:8		Mean: 47.2 months Range: 6–113 months		ACA	Positive ACA: 25.00% (5/20)	
Babińska 2017 [22]	Retrospective	ACC (20)	Mean ± SD:51.8 ± 15.6	12:8	According to the 2004 WHO classification	Range: 5–20 years	Semi-quantitative analysis (H-score)	ACC	Median H-score (25th-75th percentile): 100* (50–100)	ACC crude OR: 1.332 ACC adjusted OR: 0.811
		ACA/ACH (63)	NA	NA				ACA	Median H-score (25th-75th percentile): H-score: 100* (0–110)	
Pereira 2019 [23]	Retrospective	ACC (13)	Median:46 Range:27–59	NA	NA	NA	Morphometric computerized analysis (SA)	ACC	Mean SA ± SE: 35.97 ± 1.38%	AUC of 1.00 for a cut-off value of 27.11% stained area
		ACAn (14)	Median:49 Range:23–76	NA				ACAn	Mean SA ± SE: 16.79 ± 2.09%	

*ACA* Adrenocortical adenoma, *ACAc* Adrenocortical adenoma cortisol producing, *ACAn* Non-function adrenocortical adenoma, *ACH* Cortical nodular hyperplasia, *ACAt* Total adrenocortical adenoma, *ACC* Adrenocortical carcinoma, *AUC* Area under the curve; *H-score*: Product of the percentage of cells with positivity reactivity (0–100%) and the intensity of reactivity (0–3), *IHC*: immunohistochemistry, *NA* Non-available, *OR* Odds ratio, *SA* stained area, *SD* Standard deviation, *SE *Standard error, *s.e.m* Standard error of the mean

**Table 2:** Characteristics of the included studies regarding adrenocortical tumors proliferation index assessed by Ki-67 immunohistochemistry

First author and year	Experimental design	Sub-Groups (n)	Age (range)	Female:Male	Diagnosis tool	Follow-up time	IHC quantification method	Results		
								Group Comparisons		Diagnostic accuracy
McNicol 1997 [24]	Retrospective	ACC (40)	NA	NA	van Slooten score	Mean: 56 months Median: 86 months Range:0.5–369 months	Quantitative analysis (LI)	ACC	Median LI (range): 3.3% (0.15 to 25.1%)	NA
		ACA (14)	NA	NA		Mean: 98 months Median: 86 months Range:6–287 months		ACA	Median LI (range): 0.23% (0 to 4%)	
Arola 2000 [25]	Retrospective	ACCa (3)	NA	NA	Weiss score	NA	Quantitative analysis (LI)	ACCa	LI range: 10 to 20%	NA
		ACCc (7)	NA	NA				ACCc	LI range: 10 to 40%	
		ACCv (4)	NA	NA				ACCv	LI range: 10 to 20%	
		ACCn (13)	NA	NA				ACCn	LI range: 10 to 50%	
		ACAa (20)	NA	NA				ACAa	LI range: 1 to 2%	
		ACAc (20)	NA	NA				ACAc	LI range: 1 to 5%	
		ACAv (6)	NA	NA				ACAv	LI range: 1 to 3%	
		ACAn (15)	NA	NA				ACAn	LI range: 1 to 2%	
Gupta 2001 [26]	Retrospective	ACC (15)	Mean:48 Range:37–69	5:10	Criterion for ACC: histologic evidence of lymph node or distant organ metastases	Mean: 60 months Range: 10–160 months	Quantitative analysis (LI)	ACC	Mean LI (range): 50% (16 to 80%)	Cut-off of 10% LI with a specificity and sensitivity of 0.87
		ACA (15)	Mean:34 Range:19–60	7:8				ACA	Mean LI (range): 1.8% (0.8 to 5.6%)	
Terzolo 2001 [27]	Retrospective	ACC (11)	Mean:45.6 Range:20–62	5:6	Weiss score	At least 3 years (except for 1 patient) or until dead or disease progression	Quantitative analysis (LI)	ACC	Mean LI: 185.8 ± 60.3%	NA
		ACA (25)	Mean:39.8 Range:19–63	19:6		NA		ACA	Mean LI: 11.3 ± 16.0%	
		ACAc (6)	NA	6:0		NA		ACAc	Mean LI: 28 ± 23.6%	
		ACAn (11)	NA	7:4		NA		ACAn	Mean LI: 4.6 ± 4.8%	
		ACAa (8)	NA	6:2		NA		ACAc	Mean LI: 4.4 ± 2.6%	
Aubert 2002 [28]	Retrospective	ACC (24)	Mean:47.1 Range:19–74	17:7	ACC: presence of metastasis, gross local invasion at surgery, or local recurrence	Mean: 136.6 months Range:48–258 months	Quantitative analysis (LI)	ACC	Mean LI ± SD: 21.2 ± 18.44%	Cut-off ≥ 4 with 91.7% specificity and 95.7% sensitivity
		ACA (25)	Mean:43.2 Range:20–73	21:4				ACA	Mean LI ± SD: 2.4 ± 1.3%	
Bernini 2002 [29]	Retrospective	ACC (16)	Mean ± s.e.m: 53.4 ± 4.4 Range:19–77	7:9	ACC: tumor mass, metastasis or recurrence, mitotic ratio, necrosis, and capsule and/or vascular invasion	10 months after surgery	Quantitative analysis (LI)	ACC	Mean LI ± s.e.m: 13.7 ± 3.0%	NA
		ACAa (13)	Mean ± s.e.m: 47.4 ± 2.6 Range:27–59	5:8				ACAa	Mean LI ± s.e.m: 0.53 ± 0.08%	
		ACAn (13)	Mean ± s.e.m: 49.9 ± 2.9 Range:30–72	7:6				ACAn	Mean LI ± s.e.m: 0.53 ± 0.08%	
Giordano 2003 [30]	Retrospective	ACC (10)	NA	8:2	Weiss score	NA	Quantitative analysis (LI)	ACC	Mean LI ± SD: 8.17 ± 2.86%	NA
		ACAc (4)	NA	4:0				ACA	Mean LI ± SD: 0.95 ± 0.95%	
Kiiveri 2005 [31]	Retrospective	ACC (7/16)	Mean:56.13 Range:21–74	4:3	Weiss score	NA	Quantitative analysis (LI)	ACC	Meean LI ± SD: 26.43 ± 13.76%	NA
		ACA (17/20)	Mean:53.89 Range:26–7	13:4				ACA	Mean LI ± SD: 1.29 ± 0.47%	
Takehara 2005 [32]	Retrospecctive	ACC (3)	NA	NA	Histopathological and clinical findings	NA	Quantitative analysis (LI)	ACC	Mean LI (range): 209.4 (158.2 to 281.7%)	NA
		ACA (21)	NA	NA				ACA	Mean LI (range): 8.7 (1.4 to 33.2%)	
Schmitt 2006 [18]	Retrospective	ACC (17)	Mean ± SD:52.50 ± 16.03 Range:29–71	11:5	No uniform diagnosis criteria were used	NA	Quantitative analysis (LI)	ACC	LI > 5%: 88% (14/16) of ACC	Cut-off > 5% with a specificity of 95.5% and sensitivity of 87.5%
		ACA (22)	Mean ± SD:46.50 ± 10.79 Range:30–65	16:6				ACA	LI < 5%: 96% (21/22) of ACA	
Babinska 2008 [33]	Retrospective	ACC (11)	NA	NA	Ultrasound and CT imaging of the abdomen	1–11 years after the initial operation	Quantitative analysis (LI)	ACC	LI > 5%: 54.6% of ACC (6/11)	NA
		ACA (43)	NA	NA				ACA	LI > 5%: 2.3% of ACA (1/43)	
Szajerka 2008 [34]	Retrospective	ACC (6)	Mean: 62 Range: 50–70	3:3	NA	NA	Semi-quantitative analysis (LI x intensity)	ACC	Mean LI ± SD: 1.83 ± 1.47%	NA
		ACA (48)	Mean:52 Range:23–76	35:13				ACA	Mean LI ± SD: 0.52 ± 0.54%	
Yang 2008 [35]	Retrospective	ACCc (14)	NA	NA	Pathological and histological analysis	NA	Semi-quantitative analysis (combined staining intensity and positive cell rate)	ACCc	Negative: 14.29% of ACC (2/14) Positive: 85.71% of ACC (12/14)	NA
		ACAc (26)	NA	NA				ACAc	Negative: 92.31% of ACA (24/26) Positive: 7.69% of ACA (2/26)	
Soon 2009 [19]	Prospective	ACC (23)	NA	NA	NA	NA	Quantitative analysis (LI)	ACC	LI ≥ 5%: 70% of ACC (16/23)	AUC: 0.940
		ACA (41)	NA	NA				ACA	LI < 5%: 100% of ACA (41/41)	
Pereira 2013 [20]	Retrospective	ACC (11)	Median:46 Range:27–59	6:5	Weiss score	NA	Morphometric computerized analysis (SA)	ACC	Mean SA ± s.e.m: 2.53 ± 0.72%	ACC vs ACAt AUC: 0.96 ACC vs ACAn AUC = 0.98 ACC vs ACAc AUC = 0.94
		ACAt (20)	Median:49 Range:23–76	14:6				ACAt	Mean SA ± s.e.m: 0.08 ± 0.02%	
		ACAc (7)	NA	NA				ACAc	Mean SA ± s.e.m: 0.13 ± 0.03%	
		ACAn (13)	NA	NA				ACAn	Mean SA ± s.e.m: 0.06 ± 0.03%	
Wang 2014 [12]	Retrospective	ACC (25)	Mean:44.4 Range.18–75	14:11	Weiss score	Range:6–123 months	NA	ACC	LI > 5%: 64% of ACC (16/25)	NA
		ACA (25)	Mean:48.6 Range:34–69	14:11		Mean: 57 months Range: 6–123 months		ACA	LI > 5%: in 4% of ACA (1/25)	
Mukherjee 2015 [36]	Prospective observational	ACC (5)	NA	NA	Clinical, and biochemical evaluation, Weiss score	4 – 24 months	Semi-quantitative analysis (staining intensity x LI)	ACC	Median of Ki-67 expression: 10.50% LI > 5%: 26.3% of ACC	NA
		ACA (12)	NA	NA				ACA	Median of Ki-67 expression (range): 1.45% (0.40 to 3.6%)	
Babińska 2017 [22]	Retrospective	ACC (20)	Mean ± SD: 51.8 ± 15.6	12:8	According to the 2004 WHO classification	Median: 22.5 months Range: 5–20 years	Quantitative analysis (LI)	ACC	LI range: 0 to 11%, with a median of 1	Crude OR for diagnosis of ACC = 1.332 Adjusted OR for diagnosis of ACC = 0.811
		ACA and ACH (63)	NA	NA		Range: 5–20 years		ACA	LI range: 0 to 2%, with a median of 0	
Dalino Ciaramella 2017 [37]	Retrospective	ACC (18)	Range:25–68	13:5	Weiss score	NA	Computerized morphometric analysis (VF)	ACC	Positive VF ± SD: 0.02988 ± 0.0186	NA
		ACA (11)	Range:28–59	7:4				ACA	Positive VF ± SD: 0.00503 ± 0.0013	
Pereira 2017 [38]	Retrospective	ACC (15)	NA	NA	NA	NA	Computerized morphometric analysis (SA)	ACC	Mean SA ± s.e.m (range): 2.15 ± 0.653% (0.08–7.43)	NA
		ACAc (13)	NA	NA				ACAc	Mean SA ± s.e.m (range): 0.13 ± 0.021% (0.01–0.22)	
		ACAn (11)	NA	NA				ACAn	Mean SA ± s.e.m (range): 0.08 ± 0.028% (0.00–0.4)	
Aporowicz 2019 [39]	Retrospective	ACC (3)	Mean ± SD: 68.0 ± 14.1	2:1	NA	NA	Quantitative analysis (LI)	ACC	Mean LI ± SD: 18.66 ± 10.29%	Cut-off value of 9.73% showed a sensitivity of 84.73%, a specificity of 97.00% and AUC = 0.984
		ACA (81)	Mean ± SD: 56.7 ± 9.8	66:15				ACA	Mean LI ± SD: 4.80 ± 2.70%	
		ACAn (55)	NA	NA				ACAn	Mean LI ± SD: 4.36 ± 2.08%	
		ACAa (11)	NA	NA				ACAa	Mean LI ± SD: 6.07 ± 4.47%	
		ACAc (11)	NA	NA				ACAc	Mean LI ± SD: 5.29 ± 1.77%	
Angelousi 2021 [39]	Retrospective	ACC (24)	Median:54.5 Range:21–76	14:10	Weiss and Lin-Weiss-Bisceglia score	Median:18.4 months Range:2.12–101.9 months	Quantitative analysis (LI)	ACC	Median LI (range): 23.5% (15 to 45%)	Cut off > 5% exhibited a 95.4% specificity and 92.3% sensitivity with an AUC of 99%
		ACA (13)	Median:63.5 Range:38–71	9:4		NA		ACA	Median LI (range): 3% (1 to 5%)	
Martins-Filho 2021 [40]	Retrospective	ACC (70)	Mean ± SD: 42.1 ± 16.6	56:14	Weiss score	401 months Median:37 months (85 months for patients that did not die)	Quantitative analysis	ACC	Mean LI ± SD:: 12.4 ± 15.4% Median LI (range): 5 (0–58)	Cut-off value ≥ 3% showed a specificity of 99%, sensitivity of 57%, and AUC of 0.821
		ACA (76)	Mean ± SD: 44.2 ± 16.8	66:10		401 months		ACA	Mean LI ± SD:: 0.7 ± 1.2% Median LI: 0 (0–9)	
Maity 2022 [41]	Retrospective	ACC (9)	Mean:42 Range:27–57	2:1 (ratio)	Weiss score	Range: 4–60 months	Quantitative analysis (LI)	ACC	LI ≥ 5%: 100% of ACC (9/9) Mena LI: 11.6%	Cut-off value of 5% showed 100% specificity, sensitivity, PPV and NPV
		ACA (15)	Mean:40 Range:20–55	1.1:1 (ratio)				ACA	LI < 5%: 100% of ACA (15/15) Mean LI: 1.3%	

*ACA* Adrenocortical adenoma, *ACAa* Adrenocortical adenoma aldosterone producing, *ACAc* Adrenocortical adenoma cortisol producing, *ACAn* Non-function adrenocortical adenoma, *ACH* Cortical nodular hyperplasia, *ACAt* Total adrenocortical adenoma, *ACC* Adrenocortical carcinoma, *ACCc* Adrenocortical carcinoma cortisol producing, *ACCn* Non-function adrenocortical carcinoma, *ACCv* virilizing adrenocortical carcinoma, *AUC* Area under the curve, *CT* computerized tomography, *H-score* Product of the percentage of cells with positivity reactivity (0–100%) and the intensity of reactivity (0–3), *IHC* immunohistochemistry, *LI*: Labeling index, *PPV* Positive predictive value, *NA* Not available, *NPV* Negative predictive value, *OR* Odds ratio, *SA* Stained area, *SD* Standard deviation, s.e.m Standard error of the mean, *VF* Volume fraction

A systematic review was produced for both IGF2 and Ki-67 studies. The quantitative synthesis was only performed for Ki-67 findings, due to the high heterogeneity regarding IGF2 immunostaining evaluation among the included articles. For the meta-analysis, eligible studies evaluated and reported Ki-67 expression labelling index (LI), i.e., the percentage of Ki-67 stained nuclei in a cell population. A cut-off value of 5% was considered for the meta-analysis since it was the most widely used threshold among the included studies. For studies that did not use this threshold, they were only included in the meta-analysis if the articles presented the Ki-67 expression data for each case available for analysis, i.e., sufficient data to produce a two x two contingency table, that includes the number of true positives (TP) (ACC with a Ki-67 LI superior to 5%), false positives (FP) (ACC with a Ki-67 LI inferior to 5%), false negatives (FN) (ACA with a Ki-67 LI superior to 5%), and true negatives (TN) (ACA with a Ki-67 LI inferior to 5%). The studies that reported different cut-offs were only analyzed qualitatively due to the limited number of articles.

### Quality assessment

The methodological quality of included studies was assessed by three reviewers (SBO, MQM and SSP) using the revised Quality Assessment of Diagnostic Accuracy Studies 2 (QUADAS-2), which includes four key domains: “patient selection”, “index test”, “reference standard” and “flow and timing” [42]. Each domain comprises signaling questions, customized to suit this review (Supplementary File 2), to assist the judgment of the risk of bias, rated as high, low, and unclear. Whereas concerns of applicability, which refer to whether the study’s findings can be applied to the context of the present review, was evaluated for the patient selection, index test and reference standard. A study with a low risk of bias or low concern regarding applicability was judged as a high-quality study, whereas a report with a high-risk concerning bias and applicability, was rated as a low-quality study. For each domain, when insufficient data was provided to allow a judgment, it was rated as unclear. Any disagreements were resolved by involving a third reviewer in the discussion. The studies’ quality protocol was performed using ReviewManager (RevMan) 5.4 software (The Nordic Cochrane Centre, The Cochrane Collaboration).

### Statistical analysis

The collected values of TP, FP, FN, and TN for a cut-off value of 5% were used to calculate sensitivity, sensibility, and log diagnosis odds ratio (DOR) with 95% confidence interval (CI) for each study. The random effects model meta-analysis was adopted due to sample and diagnosis method diversity across the studies. A univariate random model was used to extract the pooled estimate of Ki-67 sensitivity, specificity, and log DOR. The results were graphically presented as forest plots with 95% CI. Additionally, the Summary Receiver Operator Characteristics (SROC) curve was constructed by plotting the “sensitivity” and “false positive rates” of each study, and curve fitting was performed using proportional hazards model approach (PHM). The AUC of SROC was used to determine Ki-67 diagnosis accuracy to discriminate ACA from ACC. AUC value ranging from 0.90 to 1.00, test was considered excellent, good for 0.80 to 0.90, poor for 0.60 to 0.70 and the test failed when AUC was below 0.60. Heterogeneity was assessed using Higggin’s I^2^, and Tau squared (τ^2^). A value I^2^ > 50% implied a substantial heterogeneity between the elegible studies [43]. Meta-analysis was conducted in R software version 4.3.0 (R Foundation for Statistical Computing, Vienna, Austria) using “meta” and “mada” packages [44].

## Results

### Search results

**Fig. 1:**
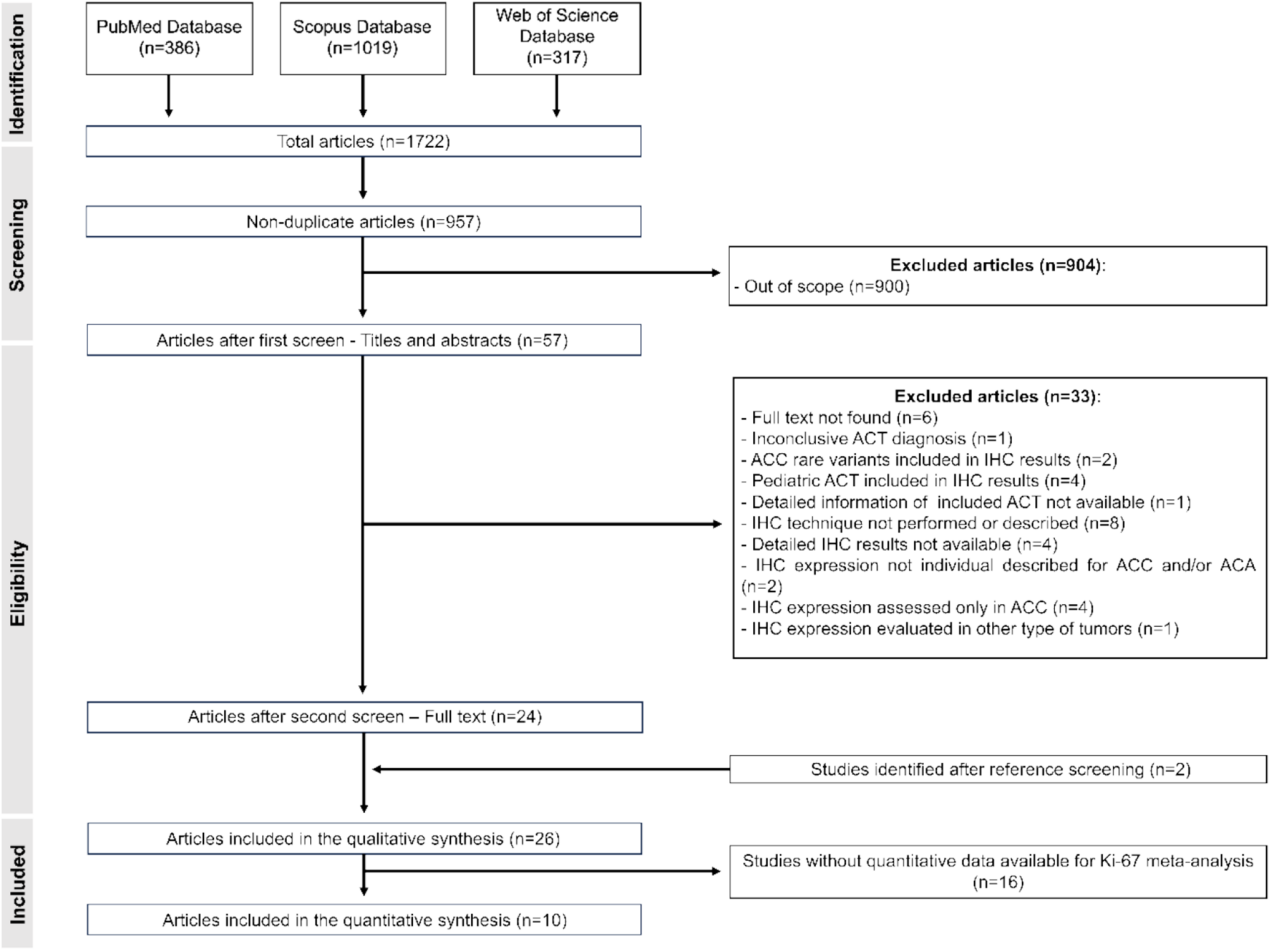
Flowchart illustrating the literature search and the selection process for the studies included in the systematic review and meta-analysis

The selection process of the studies for the qualitative and quantitative synthesis is depicted in Fig. 1. A total of 1722 articles were retrieved from a systematic search of the literature in PubMed, Scopus, and Web of Science. After elimination of duplicates (*n* = 765), 957 articles underwent title and abstract examination, resulting in the exclusion of 900 studies that were out of scope for the current review. For the remaining 54 reports, full text was collected and assessed for eligibility. A total of 33 studies were excluded due to the reasons presented in Fig. 1. Additionally, 2 studies were identified from screening references list of eligible studies. Thus, 26 studies met the pre-defined inclusion criteria and were included in the systematic review. The full text of the included studies was again reviewed in detail by two authors and, 10 reports were identified as eligible for the Ki-67 meta-analysis.

### Characteristics and quality of the studies

All original articles selected for qualitative analysis were observational studies with retrospective (*n* = 24) [12, 18, 20–39, 41, 45] and prospective (*n* = 2) data [19, 36]. The diagnosis method was identified in most of the studies including imaging modalities [21, 33], histopathological scores, including Weiss [12, 20, 21, 25, 27, 30, 31, 36, 37, 41, 45], van Sloten [24] and Lin-Weiss-Bisceglia [33, 45] scores, and the evidence of malignant features, as local invasion, or distant metastasis [26, 28, 29]. The diagnosis tool was not clearly stated or not reported in five studies [19, 23, 34, 38, 39]. Among the twenty-six studies included in the systematic review, twenty-one articles provided data for IGF2 (*n* = 2) [21, 23] or for Ki-67 (*n* = 19), in separate [24–41, 45], while five studies contained data for both markers [12, 18–20, 22]. The individual characteristics of the included studies are summarized in Table 1 and Table 2.

**Fig. 2:**
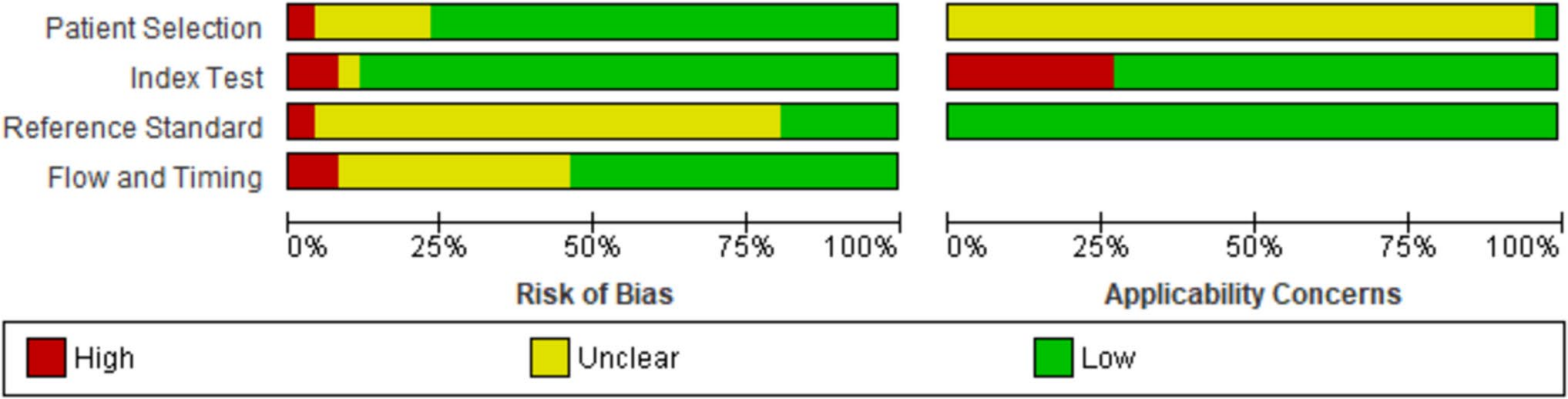
Risk of bias and applicability concern of the included studies according to Quality Assessment of Diagnostic Accuracy Studies 2 (QUADAS-2)

QUADAS-2 criteria revealed that the overall quality of the included studies was acceptable (Fig. 2), as the percentage of articles with a high risk of bias and applicability concerns did not exceed 25%. Missing information regarding blinding interpretation of IHC staining from ACT diagnosis and lack of description of ACC and ACA classification using the same diagnosis criteria were the main reasons for the unclear risk of bias in reference standard and flow and timing domains, respectively. The tumor origin was confirmed by one study through the evaluation of markers of adrenal cortical differentiation, as steroidogenic factor 1 (SF-1), melan-A and alpha-inhibin. In the remaining studies *n* = 25, this assessment was not directly reported, therefore, applicability concerns in patient selection domain were rated as unclear for most of the studies. High applicability concerns were detected for studies that performed IGF2 and/or Ki-67 IHC not aiming to assess the accuracy of these markers for ACT diagnosis. The quality assessment of each study within the four domains is presented in Supplementary File 3.

### Evaluation of IGF2 for ACC diagnosis

Solely seven out of twenty-six studies assessed IGF2 expression by IHC in both benign and malignant ACT (Table 1). IGF2 immunostaining was evaluated by three different methods: qualitative [18], semi-quantitative [12, 19, 21, 22] and quantitative analysis [20, 23].

A qualitative method was employed by one study, considering a positive staining when a perinuclear dot-like signal or Golgi pattern immunoreactivity was observed. This immunohistochemical staining pattern demonstrated a sensitivity and specificity of 76.5% and 95.5%, respectively [18].

Four studies used a semi-quantitative method to evaluate IGF2 expression, yet different score systems were employed. Two studies used a scoring system ranging from 0 to 4. Soon et al., classified a score 0–1 as negative, whereas a score ranging from 2 to 4 translated as a positive staining. This study demonstrated that 100% of ACAs were negative whereas 78% of ACCs were positive (score 2 + or more), showing a perinuclear accumulation with or without significant cytoplasmatic staining. In addition, IGF2 demonstrated to be a good marker to distinguish ACC from ACA with an AUC of 0.863 [19]. The other study found a higher number of ACC with elevated IGF2 expression when compared to ACA. Wang et al., aiming to validate the diagnostic accuracy of IGF2, evaluated its expression in 15 borderline tumors, i.e., tumors with a Weiss score of 2 or 3. However, this marker could not predict the malignant potential accurately [12]. A different scoring system was adopted by Zhu et al., comprising the combination of the percentage of cells with positive staining (score 0 to 3) and the intensity of the staining, using intensity grades of 0 (absence) to 3 (strong). IGF2 staining was observed in 25% of the benign versus 70.83% of malignant ACT cases [21]. Similarly, Babinska et al*.*, presented IGF2 expression as H-score values, which translates in the product of the percentage of cells with positive reactivity (0–100%) and the intensity of reactivity (0–3). Both ACC and ACA showed a median H-score of 100. In benign ACT, the 25th and 75th percentile range spanned from 0–110, while for malignant ACT ranged from 50 to 100. In addition, a unit increase in H-score was associated with 22% higher odds ratio of an ACC diagnosis, adjusted for age, gender, tumor size, and hormonal activity [22].

On an opposed approach, IGF2 expression was reported as the percentage of stained area quantified using a morphometric analysis tool. Pereira et al*.*, found that the percentage of IGF2 stained area was significant higher in ACC when compared to ACA, including non-functioning ACA (ACAn) and ACA with Cushing’s Syndrome (ACAc). IGF2 demonstrated to be a good marker to differentiate ACC from ACA [20]. In addition, IGF2 expression was also found to be significant higher in ACC comparing to ACAn. Indeed, this IHC marker showed an excellent discriminative power between these two entities, with 100% of sensitivity and specificity for a cut-off value of 27.1% stained area [23].

To summarize, regardless the immunostaining evaluation method adopted, the studies unanimously described the presence of IGF2 expression in most of ACC when compared to ACA, suggesting the specificity for malignant ACT. However, due to the differential immunostaining analysis methods employed within the studies, it was not feasible to conduct a meta-analysis to assess the accuracy of IGF2 in identifying malignant ACT.

### Evaluation of Ki-67 for ACC diagnosis: a descriptive approach

Tumor proliferation activity was assessed by Ki-67 IHC in benign and malignant ACT in twenty-four studies (Table 2). Most of the studies quantified Ki-67 expression proliferation index by calculating the percentage of positive cells by manual or automated count of the hot spot or random areas [22]. Specifically, this involved counting almost or at least 500 cells, minimum or about 500 [29, 45] or 1000 cells [18, 26, 28, 32], or 2000 cells [24, 40] and therefore presenting the proliferation activity of the tumors as LI. All included studies found a higher Ki-67 expression in ACC when compared to ACA [12, 18–20, 22, 24–41, 45].

Despite most of the studies presented Ki-67 LI as a continuous value, the most proposed cut-off value within the studies was 5%. Overall, the number of ACAs with a Ki-67 LI < 5% was greater when compared with ACC [18, 24–26, 28–31, 33]. For this cut-off value, the specificity and the sensitivity reported by the different studies, varied between 95.4%−100% and 87.5%−100%, respectively [18, 41, 45].

To validate the diagnostic potential of this cut-off value, Wang et al., evaluated Ki-67 expression in borderline tumors (Weiss score = 2 or 3). Among the six tumors with a Weiss score of 3, only two showed malignant behavior during follow-up, with one presenting a Ki-67 LI > 5% and the other a Ki-67 LI < 5%. The remaining patients showed no signs of disease during the follow-up, despite being classified as ACC, according to the Weiss score. However, based on Ki-67 LI (< 5%), these tumors were correctly identified as ACA. For the five borderline tumors with a Weiss score of 2 and available follow-up data, all were correctly classified by both Weiss score and Ki-67 [12]. In contrast, Schmitt et al*.*, reported two ACT that were classified as benign by the Weiss score that indeed demonstrated benign behavior during follow-up time. However, in one of these cases, Ki-67 LI was higher than 5% and so it did not support the ACA diagnosis [18]. Soon et al*.* reported an ACC with a Weiss score of 3 and Ki-67 LI < 5% that showed a benign biological behavior after 6 years of follow-up [19].

A higher threshold was setting at 10% Ki-67 LI by Gupta et al., showing a sensitivity and specificity of 87% [26]. In contrast, a higher sensitivity of 97.00% was demonstrated for a cut-off value of 9.73%, with a specificity of 84.73% and an AUC of 0.984 [39]. Five different Ki-67 thresholds—3%, 10%, 20%, 25% and 30%—were evaluated in 76 ACA and 70 ACC. Ki-67 LI ≥ 3% was able to identify the highest number of ACC (57%) when compared to the remaining Ki-67 thresholds. All the evaluated cut-offs showed high specificity ranging from 99–100%. However, various tumors classified as ACC according to the Weiss score had a Ki-67 LI < 3% (sensitivity = 57%) [40]. Indeed, all the cut-off values showed a low sensitivity varying between 14 and 57%. In contrast, Aubert et al., demonstrated that a cut-off value ≥ 4% for malignancy achieved 95.7% sensitivity and 91.7% specificity [28].

Deviating from conventional diagnostic performance measures, Babisnka et al*.*, verified that the probability of ACC diagnosis increased 0.29 times for every percentage point of Ki-67 increase. Nevertheless, this study found that Ki-67 is not an independent factor in the malignant diagnosis, since excluding the tumor size variable from the odds ratio assessment led to an overestimation of the influence of Ki-67 in ACT diagnosis [22].

In a different approach, Ki-67 expression was also presented as stained area or volume quantified using morphometric computerized analysis tools, by three studies [20, 37, 38]. Ki-67-stained area was significant higher in ACC when compared to ACA [20, 38], with an AUC value of 0.96 [20]. The authors suggested that a cut-off value of 0.50% of Ki-67-stained area was the best threshold for the differential diagnosis of ACT [20]. In addition, the same studies verified a higher AUC of 0.98 for the differential diagnosis of ACC from ACAn. Ciaramella et al., analyzed the volume fractions occupied by Ki-67 positive and negative cells (nuclei and cytoplasm) and found that the volume fraction of Ki-67 positive cells in ACC was higher than in ACA. However, no Ki-67 cut-off values, sensitivity or specificity was evaluated [37].

### Evaluation of Ki-67 for ACC diagnosis: a meta-analysis

**Fig. 3:**
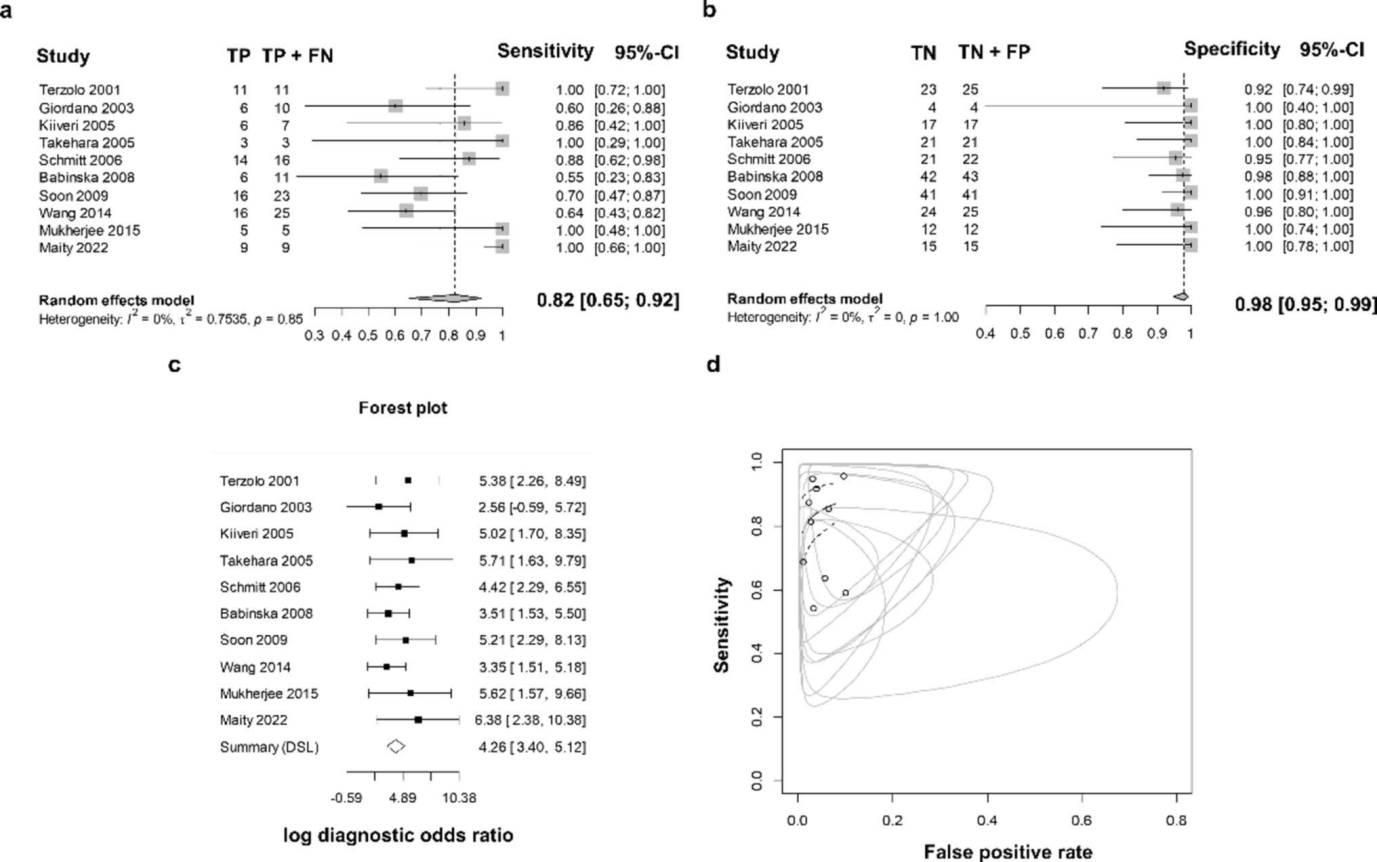
Forest plots for sensitivity (**a**), specificity (**b**) and log diagnostic odds ratio (DOR) (**c**) Summary receiver operating characteristics (SROC) curve (dashed central line) (**d**). Summary points and their confidence regions represent each included study in the analysis

The diagnostic performance of Ki-67 for the pathological discrimination between ACC and ACA was assessed at the most widely used threshold (5%) among the included studies [12, 18, 19, 27, 30–33, 36, 41]. The meta-analysis included a total of 345 ACT, from which 120 were ACC and 225 ACA. Ki-67 showed a pooled sensitivity of 0.82 (95% CI 0.65 to 0.92) (Fig. 3a) and specificity of 0.98 (95% CI 0.95 to 0.99) (Fig. 3b). Heterogeneity was not detected among the studies in terms of sensitivity (I^2^ = 0, τ^2^ = 7535, *p* = 0.85) and specificity (I^2^ = 0, τ^2^ = 0, *p* = 1.00). The log DOR varied between 2.58 (95 CI −0.59 to 5.72) and 6.38 (95 CI 2.38 to 10.38) among the studies, with a pooled value of 4.26 (95% CI 3.40 to 5.12) (Fig. 3c). Through this value, the pooled DOR value was calculated, and we found that an ACT with a Ki-67 LI superior to 5% is 70.1 times more likely to be malignant tumor. Heterogeneity was null between the studies (I^2^ = 0, τ^2^ = 0, *p* = 0.77). SROC plot displaying the summary point of each primary study in terms of sensitivity and false positive rates, together with the meta-analytic summary line (i.e., SROC curve) is present in Fig. 3d. Analyzing the SROC curve, Ki-67 for a cut-off of 5% stained cells, demonstrated to be an excellent marker for the differential diagnosis between ACA and ACC with AUC of 0.949.

### Combination of IGF2 and Ki-67 markers for ACC diagnosis

Five studies concurrently evaluated IGF2 and Ki-67 expression by IHC [12, 18–20, 22]. Nevertheless, only two studies reported on the diagnostic utility of using these markers in combination [18, 19].

One study verified that the combine use of IGF2 positive staining, characterized as a perinuclear dot-like, and a Ki-67 index > 5% was able to discriminate benign from malignant ACT with 100% sensitivity and 95.5% specificity. The combination of the two IHC markers yielded a higher sensitivity compared to IGF2 (76.5%) and Ki-67 (87.5%) alone. Whereas a specificity of 95.5% was found for each individually marker and in combination [18].

Similarly, Soon et al., demonstrated that a positive score (score 2 +) of IGF2 and/or the high Ki-67 proliferative index (≥ 5% stained cells) identified 22 of 23 ACCs (96% sensitivity) and no ACA (100% specificity). The only ACC not identified by the combination of the IHC markers, had a Weiss score of 3 and during the 6 years of follow-up, the tumor has not recurred or behaved in a malignant manner, suggesting that this case potential represents a false positive of the Weiss scoring system [19].

Both studies suggested that the combined use of IGF2 and/or Ki-67 can reliably predict the biological behavior of ACT. Of note, Soon et al., recommended the use of both two markers, particularly for tumors with a Weiss score of 2–3 with unclear malignant potential [19].

## Discussion

Distinguishing between ACA and ACC is not always easy which can cause difficulties in treatment decisions and patient follow-up. An ACT with a size superior to 4 cm and a radiological density above 10 HU, suggests malignancy, and so it is eligible for adrenalectomy [6]. After tumor removal, the differential diagnosis between ACA and ACC has relied on histopathological features comprised in scoring systems: the Weiss score, the reticulin algorithm and Lin-Weiss-Bisceglia system [1]. According to the latest clinical guidelines, the Weiss score is the most widely used and the recommended system to determine the malignant nature of ACT in adults [6]. Nevertheless, this pathological system has significant limitations: lack of reproducibility and diagnostic accuracy, particularly evident in borderline tumors, i.e., tumors with a Weiss score of 2 or 3 [11]. In the attempt to overcome the major drawbacks of the current diagnosis criteria, several immunohistochemical biomarkers have been investigated, notably IGF2 and Ki-67 [1, 8]. The present systematic review provides an in-depth overview of the existing evidence on the potential diagnosis value of IGF2 and Ki-67 for ACT. This evidence is derived from studies that assessed IGF2 and/or Ki-67 expression in both, ACC and ACA, using IHC.

*IGF2* gene encodes the growth factor IGF2, which is expressed in both fetal and adult adrenal glands. *IGF2* is one of the main oncogenes involved in ACC tumorigenesis, known to be part of a complex—IGF2 system—which activates signaling pathways, such as mitogen-activated protein kinase (MAPK), phosphatidylinositol 3-kinase (PI3K)/Akt and the mammalian target of rapamycin (mTOR) pathways, involved in proliferation, survival, and cell metastasis [23, 46–48]. Taken this in account, this marker has been pointed as a potential diagnosis marker for ACC. Indeed, IGF2 immunostaining was positive in most of ACC, in contrast to ACA [18, 19, 21]. Although positive IGF2 staining demonstrated a high specificity, only moderate sensitivity was achieved, translating the incapacity to detect all ACC [18]. IGF2 only demonstrated to be an excellent marker to differentiate ACC from ACA, when only non-functioning ACA were included [20, 23].

Together the reviewed literature suggests that IGF2 appears not to be a sensitive marker for ACC, since IGF2 expression presence and levels vary within ACC [18, 19]. Nevertheless, it is important to refer that IGF2 IHC was assessed in small cohorts of adult malignant (ranging from 11 to 25 patients) and benign (ranging from 20 to 63 patients), hence its potential lack of representativeness. In addition, different immunostaining evaluation methods were adopted among the studies: qualitative [18], semi-quantitative [12, 19, 21] and quantitative [20, 23]. This heterogeneity was the major impeding factor to assess the diagnosis accuracy of IGF2 by conducting a meta-analysis. More important, we stress the need of more studies using similar IHC evaluation methods to assess the diagnostic performance of IGF2 in ACT and alongside stratification of ACT based on functionality.

Ki-67 is a protein expressed in all cell cycle phases, except in G0, representing a cell proliferation marker that can be assessed by IHC [49]. High proliferation is a common feature of malignant tumors, and consequently Ki-67 overexpression is observed [11]. Despite nonspecific of ACC, the expression of this marker is routinely assessed by IHC in every resection specimen of ACT, for prognostication and therapeutic decisions guidance [6]. Current guidelines suggest that the cut-off value of 10% Ki-67-stained cells correlates with higher risk of recurrence, and so mitotane therapy is recommended [6, 49]. In contrast, there is no validated Ki-67 threshold value to determine the malignant nature of ACT, being the major limitation underlying the inclusion of Ki-67 as a diagnostic marker for this type of tumors. Ki-67 expression is unanimously higher in malignant compared to benign ACT [12, 18–20, 22, 24–41, 45].

The Ki-67 LI of 5% was the most widely used threshold among the evaluated studies. Yet, its diagnostic performance was evaluated in a small number of ACT (number of ACC ranging from 3 to 70; number of ACA ranging from 4 to 76). For that reason, a meta-analysis was performed to assess the diagnostic accuracy of Ki-67 marker for a cut-off value of 5% stained cells. The SROC curve revealed an AUC of 0.949, demonstrating that the Ki-67 for the studied cut-off value is an excellent marker to discriminate ACA from ACC. However, we found a pooled sensitivity of only 0.82 showing that a threshold of 5% stained is not able to identify all ACC. Nevertheless, ACT with a Ki-67 than 5% has 70.10 times more probability to have a malignant behavior, based on the pooled DOR. Our meta-analysis goes in line with reviewed literature, suggesting that different Ki-67 thresholds should be considered in future research, particularly cut-off values lower than 5% stained cells. Indeed, when different thresholds were evaluated in the same cohort, Martins-Filho et al*.*, demonstrated that a higher sensitivity was achieved for the lower cut-off value (3% Ki-67-stained cells) studied [40].

Only a minority of the included studies compared the diagnostic utility of Ki-67 to the Weiss score system [12, 18, 19]. These studies, which included tumors with follow-up data on tumor behavior, revealed that while Ki-67 correctly identified certain borderline ACT misclassified by the Weiss score, the opposite was also observed, as some ACT accurately classified as ACC or ACA by the Weiss score were not correctly diagnosed by Ki-67 LI. Thus, Ki-67 LI proved to be heterogeneous in borderline tumors, as it does not fully correlate with their benign or malignant clinical course [12, 18]. Nevertheless, the Helsinki score using Ki-67 LI as continuous values along with mitotic count and necrosis has been shown to provide a more accurate diagnosis of ACT compared to the Weiss score [1, 50]. Recently, a new histological system for ACC diagnosis was proposed comprising a set of 8 parameters, including tumor size and weight, Ki-67, mitosis, nuclear grade, atypical mitoses, invasion of capsule and necroses. Two different cut-off values were in integrated in this diagnosis system: KI-67 LI < 5% diagnosis ACA, whereas Ki-67 LI ≥ 11% diagnosis ACC. For tumors with a Ki-67 LI ranging from 5–10%, a mathematical model was created to predict the malignant potential [14].

Ki67 expression has been shown to be unevenly distributed within the tumors. Therefore, the latest clinical guidelines developed by the European Society of Endocrinology and the European Network for the Study of Adrenal Tumors, recommend that the determination of the KI-67 LI should be done on whole tumors, preferably by use an image analysis system [6]. A potential source of heterogeneity can be related to the quantification of Ki-67 expression applied within the studies. Most of the studies described Ki-67 expression as LI with variable number of cells included in the analysis, varying from 500 to 2000 cells. The Ki-67 LI was also quantified in the called hot-spots, however without information regarding the number of cells comprised in the evaluation. In addition to the areas included in the Ki-67 quantification, the use of automated systems or manual counting of Ki-67 positive nucleus can also be a source of bias. In contrast, the whole tumor was analyzed using different morphometric computerized tools, namely Ki-67 quantification of stained area [20, 23, 38]. Taken this in account, a quantification strategy comprising the whole-tumor, and the use of an automated system would contribute to a more representative and objective results, respectively.

## Limitations

To the best of our knowledge, this is the first systematic review and meta-analysis focusing on the potential diagnosis of IGF2 and/or Ki-67 to differentiate ACC from ACA in adults. A strength of our meta-analysis lies in the consistency of the inclusion criteria, which translated in null heterogeneity in the diagnostic values (sensitivity, specificity, DOR). However, some limitations of the study need to be addressed. This review could not take into consideration the IGF2 and Ki-67 IHC protocols employed by each included study, although it's worth highlighting a crucial aspect: potential variations in immunostaining results may occur due to differences in IHC protocols, such as different clones and antibodies dilutions used [51, 52]. On the other hand, IGF2 IHC staining was evaluated using different analysis methods, and taking this into consideration, a meta-analysis could not be performed. In future studies, these technical and evaluation parameters should be standardized to achieve homogeneity and enhance diagnosis accuracy of IHC technique and of immunostaining evaluation methods. Regarding the meta-analytical process, the included studies only allowed to explore the diagnostic accuracy of Ki-67 for a cut-off value of 5% stained cells, since most of the studies reported Ki-67 IHC results for this threshold value. Additionally, most of the studies did not report the individual values of Ki-67 LI for each ACT included in the studies, which interfered with the evaluation of different cut-off values. Together, these factors contributed to a restricted evaluation of the diagnostic performance of Ki-67 in ACT. As previously mentioned, the differential diagnosis between ACC and ACA is mainly based on the Weiss score. Therefore, when assessing the diagnostic utility of biomarkers, it is crucial to compare their diagnostic performance to the Weiss system. The absence of such comparative evaluation in most of the included studies contribute to the lack of evidence regarding the true diagnostic impact of IGF2 and/or Ki-67. Thus, we emphasize the need for comparative studies and the reporting of patient’s follow-up data (e.g. time of follow-up and recurrence status), as these details can provide critical insights of Weiss score misclassifications.

## Conclusion

In general, this review contributed to the understanding of the utility of IGF2 and Ki-67 immunostaining for the differential diagnosis of ACT based on the existing evidence. On the other hand, this systematic research has pointed the major limitations underlying the validation of these markers for diagnosis proposes. Indeed, IGF2 marker appears to hold a diagnostic value to identify ACC, although tumor functionality may influence its diagnostic performance. Nevertheless, studies employing similar staining analysis methods are needed to conduct a precise evaluation on the diagnosis performance of this marker. Our meta-analysis revealed that Ki-67 marker for a cut-off value of 5% stained cells, exhibited high specificity but, only moderate sensitivity, indicating its incapacity to identify all ACC. Therefore, future studies should explore different threshold values to enhance ACT diagnosis and assess whether combining the Weiss score with diagnostic markers could further refine diagnostic accuracy.

## Supplementary Information

Below is the link to the electronic supplementary material.

Supplementary file1 (DOCX 17 KB)

Supplementary file2 (DOCX 18 KB)

Supplementary file3 (DOCX 197 KB)

## Data Availability

All data is included in the manuscript.

## Abbreviations

ACA: Adrenocortical adenoma
ACAa: Adrenocortical adenoma aldosterone producing
ACAc: Adrenocortical adenoma cortisol producing
ACAn: Non-function adrenocortical adenoma
ACAt: Total adrenocortical adenoma
ACC: Adrenocortical carcinoma
ACAc: Adrenocortical carcinoma cortisol producing
ACAn: Non-function adrenocortical carcinoma
ACCv: Virilizing adrenocortical carcinoma
ACT: Adrenocortical tumors
AUC: Area under the curve
CI: Confidence interval
CT: Computerized tomography
DOR: Diagnosis odds ratio
DTA: Diagnostic test accuracy
FP: False positives
FN: False negatives
HU: Hounsfield units
IGF2: Insulin-like growth factor 2
IHC: Immunohistochemistry
LI: Labelling index
MAPK: Mitogen-activated protein kinase
mTOR: Mammalian target of rapamycin
NA: Not available
NPV: Negative predictive value
PI3K: Phosphatidylinositol 3-kinase
PPV: Positive predictive value
PRISMA: Preferred Reporting Items for Systematic Reviews and Meta-Analysis
QUADAS-2: Quality Assessment of Diagnostic Accuracy Studies 2
RevMan: ReviewManager
ROC: Receiving operating characteristic curve
SA: Stained area
SD: Standard deviation
SEM: Standard error of the mean
SF-1: Steroidogenic factor 1
SROC: Summary Receiving operating characteristic curve
TP: True positives
TN: True negatives
VF: Volume fraction

## References

[1] Mete O, et al. Overview of the 2022 WHO Classification of Adrenal Cortical Tumors. Endocr Pathol. 2022;33(1):155–96. 10.1007/s12022-022-09710-8. 35288842 10.1007/s12022-022-09710-8 PMC8920443

[2] Fassnacht M, et al. Adrenocortical carcinomas and malignant phaeochromocytomas: ESMO-EURACAN Clinical Practice Guidelines for diagnosis, treatment and follow-up. Ann Oncol. 2020;31(11):1476–90. 10.1016/j.annonc.2020.08.2099. 32861807 10.1016/j.annonc.2020.08.2099

[3] Kerkhofs TM, et al. Adrenocortical carcinoma: a population-based study on incidence and survival in the Netherlands since 1993. Eur J Cancer. 2013;49(11):2579–86. 10.1016/j.ejca.2013.02.034. 23561851 10.1016/j.ejca.2013.02.034

[4] Libe R, Huillard O. Adrenocortical carcinoma: Diagnosis, prognostic classification and treatment of localized and advanced disease. Cancer Treat Res Commun. 2023;37:100759. 10.1016/j.ctarc.2023.100759. 37690343 10.1016/j.ctarc.2023.100759

[5] Else T, et al. Adrenocortical carcinoma. Endocr Rev. 2014;35(2):282–326. 10.1210/er.2013-1029. 24423978 10.1210/er.2013-1029 PMC3963263

[6] Fassnacht M, et al. European society of endocrinology clinical practice guidelines on the management of adrenocortical carcinoma in adults, in collaboration with the european network for the study of adrenal tumors. Eur J Endocrinol. 2018;179(4):G1–46. 10.1530/EJE-18-0608. 30299884 10.1530/EJE-18-0608

[7] Lam AK. Adrenocortical carcinoma: updates of clinical and pathological features after renewed world health organisation classification and pathology staging. Biomedicines. 2021;9(2):175. 10.3390/biomedicines9020175. 33578929 10.3390/biomedicines9020175 PMC7916702

[8] Mete O, et al. Diagnostic and prognostic biomarkers of adrenal cortical carcinoma. Am J Surg Pathol. 2018;42(2):201–13. 10.1097/PAS.0000000000000943. 28877067 10.1097/PAS.0000000000000943

[9] Weiss LM, Medeiros LJ, Vickery AL Jr. Pathologic features of prognostic significance in adrenocortical carcinoma. Am J Surg Pathol. 1989;13(3):202–6. 10.1097/00000478-198903000-00004. 2919718 10.1097/00000478-198903000-00004

[10] Duregon E, et al. Pitfalls in the diagnosis of adrenocortical tumors: a lesson from 300 consultation cases. Hum Pathol. 2015;46(12):1799–807. 10.1016/j.humpath.2015.08.012. 26472162 10.1016/j.humpath.2015.08.012

[11] Vietor CL, et al. How to differentiate benign from malignant adrenocortical tumors? Cancers (Basel). 2021;13(17):4383. 10.3390/cancers13174383. 34503194 10.3390/cancers13174383 PMC8431066

[12] Wang C, et al. Distinguishing adrenal cortical carcinomas and adenomas: a study of clinicopathological features and biomarkers. Histopathology. 2014;64(4):567–76. 10.1111/his.12283. 24102952 10.1111/his.12283 PMC4282325

[13] Pohlink C, et al. Does tumor heterogeneity limit the use of the Weiss criteria in the evaluation of adrenocortical tumors? J Endocrinol Invest. 2004;27(6):565–9. 10.1007/BF03347480. 15717655 10.1007/BF03347480

[14] Urusova L, et al. The new histological system for the diagnosis of adrenocortical cancer. Front Endocrinol (Lausanne). 2023;14:1218686. 10.3389/fendo.2023.1218686. 37560295 10.3389/fendo.2023.1218686 PMC10406575

[15] Page MJ, et al. The PRISMA 2020 statement: an updated guideline for reporting systematic reviews. Rev Esp Cardiol (Engl Ed). 2021;74(9):790–9. 10.1016/j.rec.2021.07.010. 34446261 10.1016/j.rec.2021.07.010

[16] Kim KW, et al. Systematic review and meta-analysis of studies evaluating diagnostic test accuracy: a practical review for clinical researchers-part I. general guidance and tips. Korean J Radiol. 2015;16(6):1175–87. 10.3348/kjr.2015.16.6.1175. 26576106 10.3348/kjr.2015.16.6.1175 PMC4644738

[17] Salameh J-P, et al. Preferred reporting items for systematic review and meta-analysis of diagnostic test accuracy studies (PRISMA-DTA): explanation, elaboration, and checklist. BMJ. 2020;370:m2632. 10.1136/bmj.m2632. 32816740 10.1136/bmj.m2632

[18] Schmitt A, et al. IGFII and MIB1 immunohistochemistry is helpful for the differentiation of benign from malignant adrenocortical tumours. Histopathology. 2006;49(3):298–307. 10.1111/j.1365-2559.2006.02505.x. 16918977 10.1111/j.1365-2559.2006.02505.x

[19] Soon PSH, et al. Microarray gene expression and immunohistochemistry analyses of adrenocortical tumors identify IGF2 and Ki-67 as useful in differentiating carcinomas from adenomas. Endocr Relat Cancer. 2009;16(2):573–83. 10.1677/ERC-08-0237. 19218281 10.1677/ERC-08-0237

[20] Pereira SS, et al. The emerging role of the molecular marker p27 in the differential diagnosis of adrenocortical tumors. Endocr Connect. 2013;2(3):137–45. 10.1530/EC-13-0025. 23925558 10.1530/EC-13-0025 PMC3845830

[21] Zhu Y, et al. Expression of STAT3 and IGF2 in adrenocortical carcinoma and its relationship with angiogenesis. Clin Transl Oncol. 2014;16(7):644–9. 10.1007/s12094-013-1130-1. 24178245 10.1007/s12094-013-1130-1

[22] Babińska A, et al. Diagnostic and prognostic role of SF1, IGF2, Ki67, p53, adiponectin, and leptin receptors in human adrenal cortical tumors. J Surg Oncol. 2017;116(3):427–33. 10.1002/jso.24665. 28672049 10.1002/jso.24665

[23] Pereira SS, et al. IGF2 role in adrenocortical carcinoma biology. Endocrine. 2019;66(2):326–37. 10.1007/s12020-019-02033-5. 31378849 10.1007/s12020-019-02033-5 PMC6838304

[24] McNicol AM, et al. Proliferation in adrenocortical tumors: correlation with clinical outcome and p53 Status. Endocr Pathol. 1997;8(1):29–36. 10.1007/BF02739705. 12114669 10.1007/BF02739705

[25] Arola J, et al. p53 and Ki67 in adrenocortical tumors. Endocr Res. 2000;26(4):861–5. 10.3109/07435800009048609. 11196463 10.3109/07435800009048609

[26] Gupta D, et al. Value of topoisomerase II α, mib-1, p53, e-cadherin, retinoblastoma gene protein product, and her-2/neu immunohistochemical expression for the prediction of biologic behavior in adrenocortical neoplasms. Appl Immunohistochem Mol Morphol. 2001;9(3):215–21. 10.1097/00022744-200109000-00004. 11556748 10.1097/00129039-200109000-00004

[27] Terzolo M, et al. Immunohistochemical assessment of Ki-67 in the differential diagnosis of adrenocortical tumors. Urology. 2001;57(1):176–82. 10.1016/S0090-4295(00)00852-9. 11164177 10.1016/s0090-4295(00)00852-9

[28] Aubert S, et al. Weiss system revisited: a clinicopathologic and immunohistochemical study of 49 adrenocortical tumors. Am J Surg Pathol. 2002;26(12):1612–9. 10.1097/00000478-200212000-00009. 12459628 10.1097/00000478-200212000-00009

[29] Bernini GP, et al. Apoptosis control and proliferation marker in human normal and neoplastic adrenocortical tissues. Br J Cancer. 2002;86(10):1561–5. 10.1038/sj.bjc.6600287. 12085205 10.1038/sj.bjc.6600287 PMC2746588

[30] Giordano TJ, et al. Distinct transcriptional profiles of adrenocortical tumors uncovered by DNA microarray analysis. Am J Pathol. 2003;162(2):521–31. 10.1016/S0002-9440(10)63846-1. 12547710 10.1016/S0002-9440(10)63846-1 PMC1851158

[31] Kiiveri S, et al. Transcription factors GATA-6, SF-1, and cell proliferation in human adrenocortical tumors. Mol Cell Endocrinol. 2005;233(1–2):47–56. 10.1016/j.mce.2005.01.012. 15767045 10.1016/j.mce.2005.01.012

[32] Takehara K, et al. Proliferative activity and genetic changes in adrenal cortical tumors examined by flow cytometry, fluorescence in situ hybridization and immunohistochemistry. Int J Urol. 2005;12(2):121–7. 10.1111/j.1442-2042.2005.00999.x. 15733104 10.1111/j.1442-2042.2005.00999.x

[33] Babinska A, et al. The role of immunohistochemistry in histopathological diagnostics of clinically “silent” incidentally detected adrenal masses. Exp Clin Endocrinol Diabetes. 2008;116(4):246–51. 10.1055/s-2007-993164. 18393131 10.1055/s-2007-993164

[34] Szajerka A, et al. Immunohistochemical evaluation of metallothionein, Mcm-2 and Ki-67 antigen expression in tumors of the adrenal cortex. Anticancer Res. 2008;28(5 B):2959–65. 19031940

[35] Yang JY, et al. A hybrid machine learning-based method for classifying the Cushing’s Syndrome with comorbid adrenocortical lesions. BMC Genomics. 2008;9(SUPPL. 1):S23. 10.1186/1471-2164-9-S1-S23. 18366613 10.1186/1471-2164-9-S1-S23 PMC2386065

[36] Mukherjee G, et al. Histopathological study of adrenocortical masses with special references to Weiss score, Ki-67 index and p53 status. Indian J Pathol Microbiol. 2015;58(2):175–80. 10.4103/0377-4929.155308. 25885129 10.4103/0377-4929.155308

[37] Dalino Ciaramella P, et al. Analysis of histological and immunohistochemical patterns of benign and malignant adrenocortical tumors by computerized morphometry. Pathol Res Pract. 2017;213(7):815–23. 10.1016/j.prp.2017.03.004. 28554744 10.1016/j.prp.2017.03.004

[38] Pereira SS, et al. Telomerase and N-Cadherin differential importance in adrenocortical cancers and adenomas. J Cell Biochem. 2017;118(8):2064–71. 10.1002/jcb.25811. 27886397 10.1002/jcb.25811

[39] Aporowicz M, et al. Minichromosome maintenance proteins MCM-3, MCM-5, MCM-7, and Ki-67 as proliferative markers in adrenocortical tumors. Anticancer Res. 2019;39(3):1151–9. 10.21873/anticanres.13224. 30842144 10.21873/anticanres.13224

[40] Martins-Filho SN, et al. Clinical impact of pathological features including the Ki-67 labeling index on diagnosis and prognosis of adult and pediatric adrenocortical tumors. Endocr Pathol. 2021;32(2):288–300. 10.1007/s12022-020-09654-x. 33443677 10.1007/s12022-020-09654-x

[41] Maity P, et al. Diagnostic and prognostic utility of SF-1 in adrenal cortical tumours. Indian J Pathol Microbiol. 2022;65(4):814–20. 10.4103/ijpm.ijpm_153_21. 36308186 10.4103/ijpm.ijpm_153_21

[42] Whiting PF, et al. QUADAS-2: a revised tool for the quality assessment of diagnostic accuracy studies. Ann Intern Med. 2011;155(8):529–36. 10.7326/0003-4819-155-8-201110180-00009. 22007046 10.7326/0003-4819-155-8-201110180-00009

[43] Higgins JP, et al. Measuring inconsistency in meta-analyses. BMJ. 2003;327(7414):557–60. 10.1136/bmj.327.7414.557. 12958120 10.1136/bmj.327.7414.557 PMC192859

[44] Doebler PWM¨unster, Holling H. Meta-analysis of diagnostic accuracy with mada.2015. https://cran.rproject.org/web/packages/mada/vignettes/mada.pdf. Accessed Apr 2024.

[45] Angelousi A, et al. The role of immunohistochemical markers for the diagnosis and prognosis of adrenocortical neoplasms. J Personalized Med. 2021;11(3):208. 10.3390/jpm11030208. 10.3390/jpm11030208 PMC8001501 33804047

[46] Chukkalore D, et al. Adrenocortical carcinomas: molecular pathogenesis, treatment options, and emerging immunotherapy and targeted therapy approaches. Oncologist. 2024. 10.1093/oncolo/oyae029. 38381694 10.1093/oncolo/oyae029 PMC11379653

[47] Peng Y, et al. PI3K/Akt/mTOR pathway and its role in cancer therapeutics: are we making headway? Front Oncol. 2022;12:819128. 10.3389/fonc.2022.819128. 35402264 10.3389/fonc.2022.819128 PMC8987494

[48] Stefani C, et al. Growth Factors, PI3K/AKT/mTOR and MAPK signaling pathways in colorectal cancer pathogenesis: where are we now? Int J Mol Sci. 2021;22(19):10260. 10.3390/ijms221910260. 34638601 10.3390/ijms221910260 PMC8508474

[49] Mizdrak M, Ticinovic Kurir T, Bozic J. The role of biomarkers in adrenocortical carcinoma: a review of current evidence and future perspectives. Biomedicines. 2021;9(2):174. 10.3390/biomedicines9020174. 33578890 10.3390/biomedicines9020174 PMC7916711

[50] Minner S, Schreiner J, Saeger W. Adrenal cancer: relevance of different grading systems and subtypes. Clin Transl Oncol. 2021;23(7):1350–7. 10.1007/s12094-020-02524-2. 33818702 10.1007/s12094-020-02524-2 PMC8192347

[51] Koppel C, et al. Optimization and validation of PD-L1 immunohistochemistry staining protocols using the antibody clone 28–8 on different staining platforms. Mod Pathol. 2018;31(11):1630–44. 10.1038/s41379-018-0071-1. 29946185 10.1038/s41379-018-0071-1

[52] Bussolati G, Leonardo E. Technical pitfalls potentially affecting diagnoses in immunohistochemistry. J Clin Pathol. 2008;61(11):1184–92. 10.1136/jcp.2007.047720. 18326011 10.1136/jcp.2007.047720

