# Supplementary material for: The differential diagnosis of adrenocortical tumors: systematic review of Ki-67 and IGF2 and meta-analysis of Ki-67
Source: Rev Endocr Metab Disord. 2025 Jan 31;26(2):261–78. doi: 10.1007/s11154-025-09945-w (PMC11920293; doi:10.1007/s11154-025-09945-w)
Supplement: Supplementary file 1 — Supplementary file1 (DOCX 17 KB) [file 11154_2025_9945_MOESM1_ESM.docx]

**Reviews in Endocrine and Metabolic Disorders**

**Title:** The Differential Diagnosis of Adrenocortical Tumors: Systematic Review of Ki-67 and IGF2 and Meta-analysis of Ki-67

**Authors:** Sofia B. Oliveira^1,2,3,4^, Mariana Q. Machado^1,2^, Diana Sousa^1,2,5^, Sofia S. Pereira^1,2*^, Duarte Pignatelli^1,2,3,4,6, *^

**Affiliations:**

^1^UMIB – Unit for Multidisciplinary Research in Biomedicine; ICBAS – School of Medicine and Biomedical Sciences, University of Porto, Porto, Portugal

^2^ITR – Laboratory for Integrative and Translational Research in Population Health, Porto, Portugal

^3^i3S – Institute for Research and Innovation in Health, University of Porto; IPATIMUP – Institute of Molecular Pathology and Immunology of the University of Porto, Porto, Portugal

^4^Department of Endocrinology, Unidade Local de Saúde de São João, Porto, Portugal

^5^UCP – Universidade Católica Portuguesa, Faculdade de Medicina Dentária, Viseu, Portugal

^6^Department of Biomedicine, Faculty of Medicine, University of Porto, Porto, Portugal

* Sofia S. Pereira and Duarte Pignatelli equally contributed to this work.

**Corresponding author:** Sofia S. Pereira (sspereira@icbas.up.pt), PhD, Assistant Professor at School of Medicine and Biomedical Sciences (ICBAS)

**Supplementary File 1- Database search**

PubMed:

("adrenocortical" OR "adrenal") AND (“carcinoma*" OR "cancer*" OR "neoplasm*" OR "malignant" OR “tumo*” OR "adenoma*” OR “benign” OR "ACC" OR "ACA" OR "ACT") AND ("KI-67” OR “MIB-1" OR "MIB1" OR "KI67" OR “IGF2” OR “IGF-2” OR "Insulin-like growth factor 2") AND ("immunohisto*” OR “expression” OR "diagnosis") NOT (“pediatric” OR “infant*” OR “child*”)

Scopus:

TITLE-ABS-KEY ("adrenocortical" OR "adrenal") AND (“carcinoma*" OR "cancer*" OR "neoplasm*" OR "malignant" OR “tumo*” OR "adenoma*” OR “benign” OR "ACC" OR "ACA" OR "ACT") AND TITLE-ABS-KEY ("KI-67” OR “MIB-1" OR "MIB1" OR "KI67" OR “IGF2” OR “IGF-2” OR "Insulin-like growth factor 2") AND ("immunohisto*” OR “expression” OR "diagnosis") AND NOT TITLE-ABS-KEY (“pediatric” OR “infant*” OR “child*”)

Web of Science:

#1: ((TS=(“adrenocortical” OR “adrenal”)) NOT (TS=("pediatric" OR "infant*" OR "child*")))

*Indexes=SCI-EXPANDED, SSCI, A&HCI, CPCI-S, CPCI-SSH, ESCI, CCR-EXPANDED, IC Timespan=All years*

AND

#2: ((TS=("carcinoma*" OR "cancer*" OR "neoplasm" OR "malignant" OR "tumo*" OR "adenoma*" OR “benign” OR "ACC" OR "ACA" OR "ACT")))

*Indexes=SCI-EXPANDED, SSCI, A&HCI, CPCI-S, CPCI-SSH, ESCI, CCR-EXPANDED, IC Timespan=All years*

AND

#3: ((TS=("KI-67" OR "MIB-1" OR "MIB1" OR "KI67" OR "IGF2" OR "IGF-2" OR "Insulin-like

growth factor 2")))

*Indexes=SCI-EXPANDED, SSCI, A&HCI, CPCI-S, CPCI-SSH, ESCI, CCR-EXPANDED, IC Timespan=All years*

AND

#4: ((TS=("immunohisto*" OR “expression” OR "diagnosis")))

*Indexes=SCI-EXPANDED, SSCI, A&HCI, CPCI-S, CPCI-SSH, ESCI, CCR-EXPANDED, IC Timespan=All years*

AND

#5: (LA=(English)) AND DT=(Article)

*Indexes=SCI-EXPANDED, SSCI, A&HCI, CPCI-S, CPCI-SSH, ESCI, CCR-EXPANDED, IC Timespan=All years*

Final Search (6): #5 AND #4 AND #3 AND #2 AND #1
