# Supplementary material for: The differential diagnosis of adrenocortical tumors: systematic review of Ki-67 and IGF2 and meta-analysis of Ki-67
Source: Rev Endocr Metab Disord. 2025 Jan 31;26(2):261–78. doi: 10.1007/s11154-025-09945-w (PMC11920293; doi:10.1007/s11154-025-09945-w)
Supplement: Supplementary file 3 — Supplementary file3 (DOCX 197 KB) [file 11154_2025_9945_MOESM3_ESM.docx]

**Reviews in Endocrine and Metabolic Disorders**

**Title:** The Differential Diagnosis of Adrenocortical Tumors: Systematic Review of Ki-67 and IGF2 and Meta-analysis of Ki-67

**Authors:** Sofia B. Oliveira^1,2,3,4^, Mariana Q. Machado^1,2^, Diana Sousa^1,2,5^, Sofia S. Pereira^1,2*^, Duarte Pignatelli^1,2,3,4,6, *^

**Affiliations:**

^1^UMIB – Unit for Multidisciplinary Research in Biomedicine; ICBAS – School of Medicine and Biomedical Sciences, University of Porto, Porto, Portugal

^2^ITR – Laboratory for Integrative and Translational Research in Population Health, Porto, Portugal

^3^i3S – Institute for Research and Innovation in Health, University of Porto; IPATIMUP – Institute of Molecular Pathology and Immunology of the University of Porto, Porto, Portugal

^4^Department of Endocrinology, Unidade Local de Saúde de São João, Porto, Portugal

^5^UCP – Universidade Católica Portuguesa, Faculdade de Medicina Dentária, Viseu, Portugal

^6^Department of Biomedicine, Faculty of Medicine, University of Porto, Porto, Portugal

* Sofia S. Pereira and Duarte Pignatelli equally contributed to this work.

**Corresponding author:** Sofia S. Pereira (sspereira@icbas.up.pt), PhD, Assistant Professor at School of Medicine and Biomedical Sciences (ICBAS)

**Supplementary File 3 – Assessment of the methodological quality and risk of bias of the studies included using Quality Assessment of Diagnostic Accuracy Studies (QUADAS-2)**


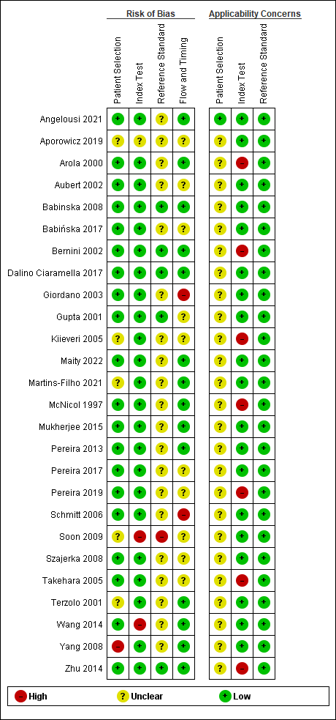


**Supplementary Figure 1 –** Summary of methodological quality of studies according to the Quality Assessment of Diagnostic Accuracy Studies (QUADAS-2) score.
